# Laparoscopic adjustable gastric banding: a report of 228 cases

**DOI:** 10.1093/gastro/got023

**Published:** 2013-08-10

**Authors:** Xin Wang, Cheng-zhu Zheng, Xu-sheng Chang, Xin Zhao, Kai Yin

**Affiliations:** Department of General Surgery, Changhai Hospital, Shanghai, China

**Keywords:** obesity, gastric banding, laparoscopy, complications, percentage of excess weight loss

## Abstract

**Objective:** To evaluate the surgical outcomes and complications after laparoscopic adjustable gastric banding (LAGB) in obese patients.

**Methods:** This retrospective study included 228 patients (73 males and 155 females, mean age, 32.5 ± 10.3 years) who underwent LAGB at the Changhai Hospital of the Second Military Medical University from June 2003 to June 2011. The body weight and postoperative complications were followed up.

**Results:** The pre-operative mean body mass index (BMI) was 39.5 ± 6.3 kg/m^2^. Except in one case of inadequate exposure of the stomach, all laparoscopic procedures were successfully accomplished, with no conversion to open surgery. The mean operation time was 65.0 ± 20.3 min. The mean hospital stay was 2.7 ± 0.9 days. Early postoperative complications (<30 days) occurred in five cases (2.2%) and late complications (>30 days) occurred in 75 cases (32.9%), including 56 cases (24.6%) with band-associated complications. The percentage of excess weight loss (EWL%) at 1, 3 and 5 years was 40.5 ± 30.5%, 59.5 ± 41.5% and 58.9 ± 46.4%, respectively. The percentages of patients with EWL% >25%, >50% and >75% were, respectively, 60%, 33% and 0% at 1 year follow-up, 43%, 39%, and 16% at 3 years follow-up and 40%, 34% and 16% at 5 years follow-up.

**Conclusion** Although LAGB has low peri-operative mortality and morbidity rates, it is associated with a high late complication rate and unsatisfactory weight loss. It may be optional, but not the first choice, for the treatment of obesity.

## INTRODUCTION

With social development and changes in diet, obesity has become a major public health problem worldwide, with increasing incidence. Currently, surgical treatment is considered to be the only effective method for long-term control of body weight. Laparoscopic adjustable gastric banding (LAGB) has been one of the first and most popular surgical methods to treat simple obesity worldwide. LAGB is widely used because it is a simple surgical procedure and associated with a low surgical risk [1]. In the present study, we retrospectively investigated 228 obese patients, who underwent LAGB at the Changhai Hospital of the Second Military Medical University from June 2003 to June 2011. The purpose of this study was to evaluate the surgical outcomes and complications after LAGB in obese patients.

## MATERIALS AND METHODS

### Patients

This study included 228 obese patients (73 males and 155 females), who underwent LAGB from June 2003 to June 2011. Their average age was 32.5 ± 10.3 years (range: 14–65 years). Their mean body weight was 113.5 ± 20.4 kg (range: 72–188 kg). Mean body mass index (BMI) was 39.5 ± 6.3 kg/m^2^ (range: 28–57.2 kg/m^2^). All patients had undergone conservative treatments, which failed to reduce body weight. Prior to the operation, all patients were examined in the Departments of Endocrinology, Anesthesia and Nutrition to exclude contra-indications to surgery. The clinical data were collected, including patient’s demographics, routine examinations, pre-operative examinations, pre-operative comorbidities and early postoperative complications. Patients were followed up for periods of 3–70 months. Their BMI, percentage of excess weight loss (EWL%), late complications, band replacement or removal and conversion of band to other surgical approaches were followed up. EWL% was calculated using the normal BMI (<23 kg/m^2^) for the Asian population [2].

### Surgical procedures

All patients had placement of gastric tube and urinary catheter before operation. Under general anesthesia, patients were placed in a reverse Trendelenburg position with 70 degrees of abduction of the lower extremities. Prophylactic intravenous antibiotics were administered during induction of anesthesia. The four- or five-port method was used for LAGB. The pneumoperitoneum pressure was set to 15 mmHg. Swedish Adjustable Gastric Bands (SAGB) were used for all patients. After retraction of the left lobe of the liver and exposure of the left border of the gastric cardia, the superficial serosa at the angle of His was dissected form the left crus of diaphragm to expose the lesser omentum. After the avascular zone of the hepatogastric ligament was cut open, the dissection continued toward the cardiac notch at the posterior wall of the stomach. The retrogastric tunnel was created behind the stomach using the Goldfinger™ dissector (Obtech, Ethicon Endo-Surgery) by passing from the posterior wall of the lesser curvature to the loose serosa at the angle of His. After expansion of the main operating port (located in the upper abdomen) to 1.5 cm in diameter, the band was inserted into the abdominal cavity, grasped by the Goldfinger and passed through the retrogastric tunnel. Both ends of the band were jointed and secured outside the gastric wall below the cardia of the stomach. The seromuscular layer at the greater curvature of the stomach was fixed to the left crus of diaphragm with sutures. Two or three seromuscular sutures, above and below the band, were applied to fix the band to the anterior wall of the stomach. The band catheter was withdrawn from the abdominal cavity via the main operating port. After the port incision was expanded to 3–4 cm in diameter to expose the external oblique fascia, the band catheter was connected to an adjustable pump, which was subsequently fixed on the surface of the external oblique fascia. All incisions were closed.

### Postoperative management and follow-up

Antibiotics were used routinely to prevent postoperative infection. On the second postoperative day, patients underwent upper gastro-intestinal radiography by taking iodine contrasts. Management included a clear liquid diet on the second postoperative day, followed by full liquids from the third postoperative days to the fourth week. The first band fill started on the first month postoperatively with the injection volume <4 ml (typically 2 ml) under the supervision of X-ray. Based on change in body weight, the injection volume increased by 0.5–1 ml on each occasion and the total injection volume was less than 9 ml. The patients were regularly followed up. The patient’s particulars were examined, including body weight, waist circumference, hip circumference, diet and quality of life. Upper gastrointestinal radiology or endoscopy was performed if the body weight loss was abnormal.

### Statistical analysis

All statistical analysis was conducted using SPSS 19.0 version (SPSS Inc., Chicago. IL). Data are presented as 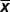 ± s. Paired *t*-tests were used to compare the differences between the pre-operative and postoperative data. Probability values less than 0.05 were considered statistically significant.

## RESULTS

### Postoperative complications

A total of 228 patients underwent LAGB. Except in one case of inadequate exposure of the stomach, all laparoscopic procedures were successfully accomplished, with no conversion to open surgery. The mean operation time was 65.0 ± 20.3 min (range: 45–185 min). The mean hospital stay was 2.7 ± 0.9 days (range: 2–6 days). No deaths occurred during the peri-operative period. Early postoperative complications (<30 days) occurred in five cases (2.2%) due to port site infection. All patients were healed after local dressing changes and treatment with antibiotics.

Late complications (>30 days) occurred in 75 cases (32.9%) (Table 1). Band-associated complications occurred in 56 cases (24.6%). Port site infection and replacement or repositioning of the port occurred in seven cases and was relieved after local incision of the port site and readjustment of the port. Replacement or repositioning of the band occurred in six cases. Band readjustment was carried out in two cases and band removal was performed in four cases with satisfactory weight loss. Dysphagia occurred in 13 cases and was relieved after adjustment of the band tightness. Gastric band erosion occurred in 28 cases. In 22 cases, the band was surgically removed, followed by repair of the gastric wall. For the other six cases, no specific treatment was carried out. Chronic gastrointestinal obstruction occurred in two cases and was treated with laparoscopic exploration to remove the adhesions. Non-band-associated complications occurred in 19 cases (8.3%), including hair loss in 10 cases and anemia in nine cases. The symptoms were relieved after nutritional support.

**Table 1:** Late complications in 228 patients after LAGB

Late complications	N (%)
Band-associated complications	56 (24.6)
Port site infection and replacement or repositioning of the port	7 (3)
Replacement or repositioning of the band	6 (2.6)
Dysphagia	13 (5.7)
Gastric band erosion	28 (12.3)
Chronic gastrointestinal obstruction	2 (0.9)
Non- band-associated complications	19 (8.3)
Hair loss	10 (4.4)
Anemia	9 (3.9)
Total	75 (32.9)

### Weight loss

**Figure 1:**
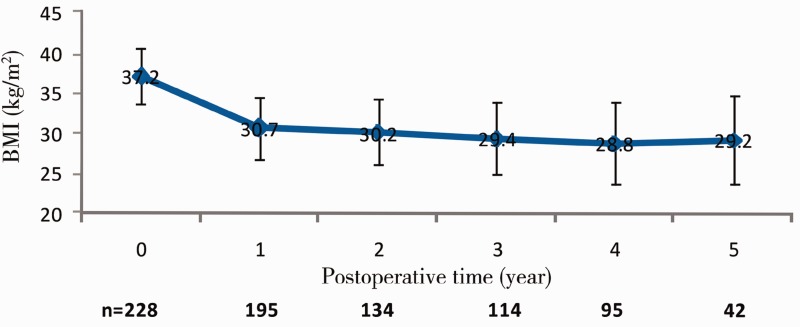
Changes in BMI in 228 patients at 1, 2, 3, 4 and 5 years after LAGB.

**Figure 2:**
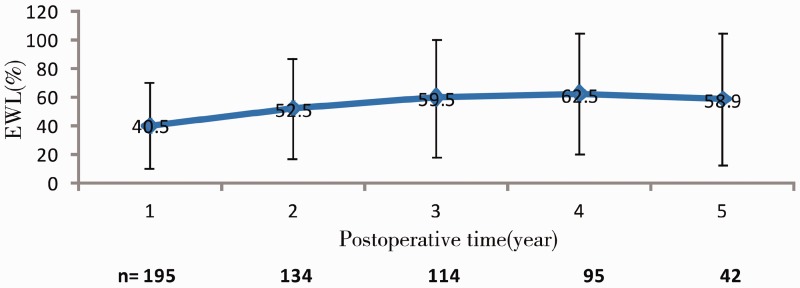
Changes in EWL% in 228 patients at 1, 2, 3, 4 and 5 years after LAGB.

For the rate of loss of follow-up, not all the patients have finished the 5-year follow-up. There were 195, 134, 114, 95 and 42 patients followed up at 1, 2, 3, 4 and 5 years after surgery, respectively. The body weight, BMI and EWL% of these patients are shown in Table 2. The body weight and BMI decreased with an increase in the follow-up period. Compared with pre-operative body weight and BMI, the postoperative body weight and BMI were significantly reduced (Table 2 and Fig. 1). The EWL% increased with the increase in the follow-up period (Table 2 and Fig. 2).

**Table 2:** Body weight, BMI and EWL% in 228 patients after LAGB

Follow-up period	*n*	Body weight (Kg)			BMI(kg/m^2^)			EWL%
		Mean ± SD	*t*-value	*P*-value	Mean ± SD	*t*-alue	*P*-value	
Pre-operative	228	113.5 ± 20.4	–	–	39.5 ± 6.3	–	–	–
Postoperative								
1 year	195	94.5 ± 26.4	10.31	<0.05	30.7 ± 3.9	10.47	<0.05	40.5 ± 30.5
2 years	134	91.5 ± 24.5	9.58	<0.05	30.2 ± 4.1	10.46	<0.05	52.5 ± 35.4
3 years	114	88.3 ± 22.8	10.32	<0.05	29.2 ± 4.5	11.43	<0.05	59.5 ± 41.5
4 years	95	83.2 ± 27.3	11.35	<0.05	28.8 ± 5.1	12.13	<0.05	62.5 ± 42.5
5 years	42	86.2 ± 26.4	10.96	<0.05	29.2 ± 5.6	12.28	<0.05	58.9 ± 46.4

EWL% = [body weight loss (kg) / the part of pre-operative body weight (kg) with BMI >23] × 100%

**Figure 3:**
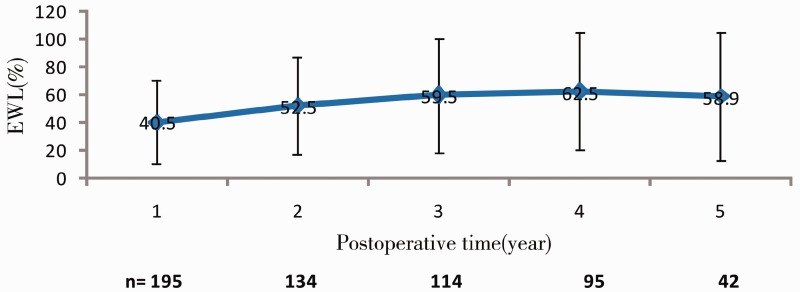
The distribution of EWL% in 228 patients at 1, 2, 3, 4 and 5 years after LAGB.

At 1 year follow-up, 64 patients (32.8%) had EWL% >50%, but none of them had EWL% >75%. At 3 years follow-up, 62 patients (54.4%) had EWL% >50%, including 18 patients (15.8%) with EWL% >75. At 5 years follow-up, 23 patients (54.8) had WEL >50%, including 9 patients (21.4%) with EWL% >75 (Fig. 3).

## DISCUSSION

Obesity has become a serious public health issue worldwide. It has been reported that in 35–74-year-old Chinese adults, approximately 26.9% of men and 31.1% of woman are obese [3]. The incidence of obesity has been increasing and the correlation of obesity with metabolic diseases is also increasingly recognized worldwide. Since the first report about the surgical treatment of obesity in 1954, several surgical procedures has developed, including jejunoileal bypass, gastric bypass, Roux-en-Y gastric bypass, biliopancreatic diversion, biliopancreatic diversion with duodenal switch, vertical banded gastroplasty, adjustable gastric banding, mini-gastric bypass, sleeve gastrectomy, duodenum-jejunal bypass and ileal transposition. Although there are many surgical procedures for the treatment of obesity, they can be classified into three categories based on changes on the gastrointestinal tract: (i) surgical procedures that result in restriction of food intake, (ii) surgical procedures that reduce the absorption of nutrients and (iii) surgical procedures that restrict food intake and reduce the absorption of nutrients. Since the introduction of LAGB in 1993, it has gained worldwide popularity, especially in North America and Europe, probably because it is easy to perform, less invasive and reversible [4]. In 2003, our department carried out China's first LAGB for the treatment of obesity. With the development of LAGB in China, the experience of the technique in the treatment of obesity in Chinese population has increased.

With the increase in the number of procedures and the follow-up period in obese patients after LAGB, a new comprehensive re-evaluation has been made worldwide. It has been reported that LAGB has low mortality and early complication rates, compared with other surgical procedures, in the treatment of obesity [5]. Consistent with the previous report, in the present study we found that the early complication rate was as low as 3.3% and the peri-operative mortality rate was 0%. However, recent studies have shown that LAGB has a high late complication rate [6]. Boza *et al.* reported that the late complication rate was 33.6%, which is similar to 32.9% in the present study [7]. In addition, we found that the band-associated late complication rate was 24.6%. Gastric band erosion occurred in 28 cases (12.3%), which was much higher than that (1.46%) reported by Egberts *et al.* [8]. In the present study, all 28 patients with gastric band erosion were not regularly followed up to adjust the band, suggesting that gastric band erosion was due to long-term band compression on the gastric wall. It has been reported that band erosion into the gastric wall is associated with surgical skills [9]. However, in the present study, all operations were performed by the same surgeon and the erosion rate did not increase over the follow-up period. In addition, based on the follow-up endoscopic and laparoscopic results, gastric perforation at the band closure site occurred in 20 cases, suggesting that gastric peroration was associated with unevenly distributed band pressure on the gastric wall and rough band surface at the band closure site. The high late complication rate in this study may be associated with the failure of the follow-up to readjust the band in our hospital because of geographic and social factors and inability to detect the complications in the local hospital. Lin *et al.* reported that band re-adjustment during the follow-up period significantly influences the effects of LAGB on weight loss [10].

In China, EWL% was previously calculated, based on the definition of obesity as BMI ≥ 25 kg/m^2^ [11]. Because there is an obvious difference in body weight between Asian and European people, obesity in the Asian population is currently defined as BMI ≥ 23 kg/m^2^ according to the 2000 Asia-Pacific Workshop on Obesity. In the present study, we used BMI ≥ 23 kg/m^2^ as the definition of overweight to calculate the EWL%. In this way, EWL% can objectively reflect body weight change in Asian people. In a retrospective study, LAGB has been reported to be associated with a high late complication rate and an unsatisfactory weight loss [12]. Because several factors—such as the follow-up examination after LAGB, control of diet and psychological status of the patients—contribute to the effect of LAGB on weight loss, weight loss in patients after LAGB is unstable. Boza *et al.* reported that EWL% at 5 years follow-up was 58.4% [7]. Similarly, O’Brien *et al.* found that EWL% was 54.2% in obese patients with a long follow-up period after LAGB [13]. In the present study, we found that the mean EWL% reached to >50% at 2 years follow-up, but only 32.8%, 54.4% and 54.8% of patients had EWL% > 50% at 1, 3 and 5 years follow-up, respectively. Therefore, although the percentage of patients with EWL% >25% is very high, the percentage of patients with EWL% >50, and particularly >75%, is very low. Therefore, the long-term weight loss after LAGB is not satisfactory in the present study.

LAGB has been demonstrated to have some advantages, such as ease of operation, low risk and low early complication rate. However, LAGB is associated with a high late complication rate and a requirement of a high level of follow-up management. Although LAGB is efficient in reducing body weight in obese patients, the percentage of patients with successful and perfect weight loss is unsatisfactory. With advances in laparoscopic skills and improvement in other surgical procedures, there is a trend towards replacing LAGB in the treatment of obesity [14]. We believe that LAGB should not be the first choice for the treatment of obesity, but should remain as a therapeutic alternative for obese patients, such as the young and patients who do not tolerate removal of the gastrointestinal tract.

**Conflict of interest:** none declared.
